# Complete mitochondrial genome of *Pseudachorutes palmiensis* (Collembola: Neanuridae)

**DOI:** 10.1080/23802359.2019.1704187

**Published:** 2020-01-07

**Authors:** Jie Dong, Feng Zhang, Xingliang Wang

**Affiliations:** Department of Entomology, College of Plant Protection, Nanjing Agricultural University, Nanjing, China

**Keywords:** Neanuridae, gene order, mitogenome, phylogeny

## Abstract

We assembled one mitochondrial genome of *Pseudachorutes palmiensis* from Illumina sequencing data. The circularized mitochondrial assembly is 17,110 bp in length, including 13 protein-coding genes, 22 transfer RNA genes, and two ribosomal RNA genes, which showed the typical insect mitochondrial gene composition, but had different order with most springtails. The overall base composition is 33.5% for A, 31.7% for T, 22.2% for C, and 12.5% for G. A phylogeny of 10 collembolans showed *P. palmiensi* was clustered within Neanuridae.

The genus *Pseudachorutes* Tullberg, the second largest genus of Neanuridae Börner (Poduromorpha), have 119 species recorded (Bellinger et al. 1996‒2019) all over the world. Only few short mitochondrial and ribosomal sequences have been reported for *Pseudachorutes* to date (NCBI, accessed 2019 Oct 28). Here we assembled the mitochondrial genome of *P*. *palmiensis* Börner, which can be helpful for assessing its systematic position.

Voucher specimen of *P. palmiensis* was collected from 24-Boulazac, France (45.154°N, 0.770°E; NCBI BioSample accession SAMN11866858; specimen Accession number 24-066). Total genomic DNA was extracted from whole body of the individual, using the QIAamp DNA Micro Kit (Qiagen, GmbH, Germany) and sequenced by NovaSeq 6000, generating 9.07 Gbp of clean reads (NCBI SRA accession SRR9131240). The voucher specimen and corresponding extracted DNA were stored at −20 °C in the Nanjing Agricultural University, Nanjing, China. The mitogenome was assembled with NOVOPlasty v2.7.0 (Dierckxsens et al. 2017), annotated with MitoZ v2.4 (Meng et al. 2019), and deposited in GenBank with an accession number MN660051.

The circularized mitochondrial assembly of *P. palmiensis* was 17,110 bp in length, including 13 protein-coding genes, 22 transfer RNA genes, and two ribosomal RNA genes. The gene composition is similar with most springtails, such as *Sinella curviseta* (NC_042755.1) and *Friesea grisea* (NC_010535.1), but had different order. Genes located between tRNA^Glu^ and ND1 are tRNA^Thr^, ND6, CYTB, tRNA^Ser^, tRNA^Phe^, ND5, tRNA^His^, ND4, ND4L and tRNA^Pro^ instead of tRNA^Phe^, ND5, tRNA^His^, ND4, ND4L, tRNA^Thr^, tRNA^Pro^, ND6, CYTB and tRNA^Ser^. The mean length of tRNAs is 66 bp, ranging from 64 bp to 73 bp. The overall base composition was 33.5% for A, 31.7% for T, 22.2% for C, and 12.5% for G, demonstrating a bias of higher AT content (65.2%) than GC content (35.8%). Nine PCGs used TAA as the stop codon, one PCG (ND4) used TAG and the other three (ND5, CYTB and ND6) used uncompleted T and TA.

Eleven amino acid sequences were aligned using MAFFT v7.407 (Katoh and Standley 2013) and trimmed with trimAl v1.4.1 (Capella-Gutiérrez et al. 2009) with the heuristic method ‘automated1’. And the phylogeny was constructed from 11 sequences including *P. palmiensis*, the other nine Poduromorpha species and one Entomobryomorpha specie as an outgroup, using IQ-TREE v1.6.10 (Nguyen et al. 2015) with the partitioning model. Monophyly of Neanuridae was recovered because *P. palmiensis* was clustered within Neanuridae (Figure 1).

**Figure 1.**
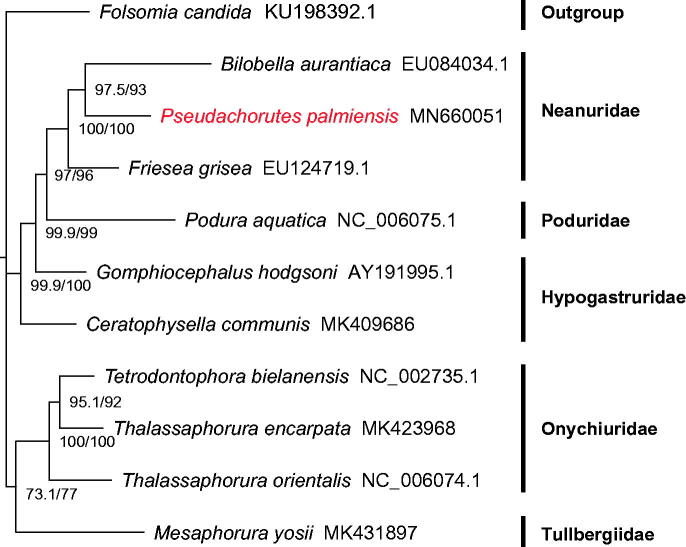
Maximum likelihood phylogenetic tree inferred from 13 PCGs. SH-aLRT and UFBoot support values are given on nodes.
